# Non-Coding RNAs in Legumes: Their Emerging Roles in Regulating Biotic/Abiotic Stress Responses and Plant Growth and Development

**DOI:** 10.3390/cells10071674

**Published:** 2021-07-02

**Authors:** Uday Chand Jha, Harsh Nayyar, Nitin Mantri, Kadambot H. M. Siddique

**Affiliations:** 1 ICAR—Indian Institute of Pulses Research (IIPR), Kanpur 208024, India; 2 Department of Botany, Panjab University, Chandigarh 160014, India; harshnayyar@hotmail.com; 3 School of Science, RMIT University, Melbourne 3083, Australia; nitin.mantri@rmit.edu.au; 4 The UWA Institute of Agriculture, The University of Western Australia, Perth 6001, Australia

**Keywords:** ncRNA, miRNA, lncRNA, biotic stress, abiotic stress, gene

## Abstract

Noncoding RNAs, including microRNAs (miRNAs), small interference RNAs (siRNAs), circular RNA (circRNA), and long noncoding RNAs (lncRNAs), control gene expression at the transcription, post-transcription, and translation levels. Apart from protein-coding genes, accumulating evidence supports ncRNAs playing a critical role in shaping plant growth and development and biotic and abiotic stress responses in various species, including legume crops. Noncoding RNAs (ncRNAs) interact with DNA, RNA, and proteins, modulating their target genes. However, the regulatory mechanisms controlling these cellular processes are not well understood. Here, we discuss the features of various ncRNAs, including their emerging role in contributing to biotic/abiotic stress response and plant growth and development, in addition to the molecular mechanisms involved, focusing on legume crops. Unravelling the underlying molecular mechanisms and functional implications of ncRNAs will enhance our understanding of the coordinated regulation of plant defences against various biotic and abiotic stresses and for key growth and development processes to better design various legume crops for global food security.

## 1. Introduction

**Figure 1:**
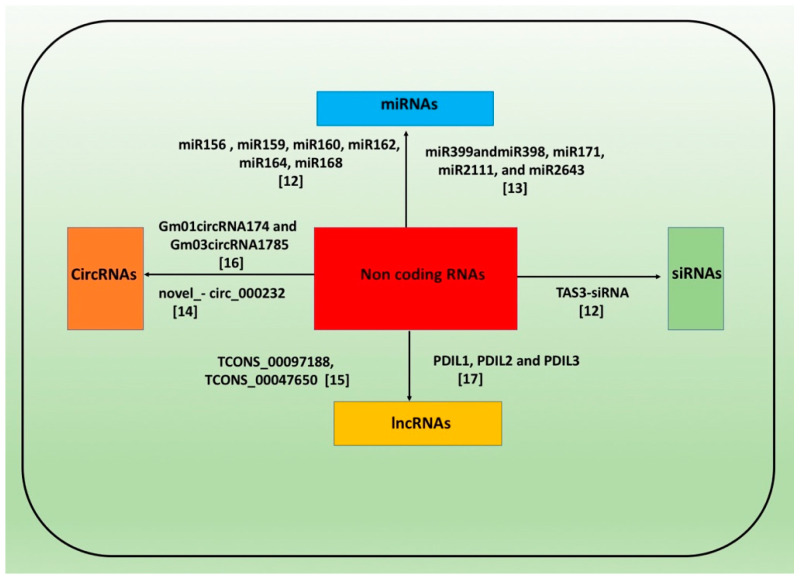
Example of major classes of ncRNA regulation for growth and development processes and stress tolerance in legume plants [12,13,14,15,16,17].

Legumes are the third largest family of flowering plants, and grain legumes are essential components of the human food diet, supplying ‘plant-based dietary proteins’ and essential micronutrients and vitamins [1,2,3]. Thus, legume crops serve as an essential component for sustaining global food security. Their ability to fix atmospheric nitrogen through symbiotically active bacteria in root nodules enriches soil nitrogen content and minimizes the use of chemical-based nitrogenous fertilizers, thus protecting the environment from pollution [1]. In the past, elucidating the function of protein-coding genes controlling biotic and abiotic stresses and developmental processes in plants has involved conventional breeding and biochemical and molecular approaches [4]. However, rapid progress in functional genomics, especially transcriptome sequencing by RNA-seq, has given us the opportunity to investigate RNAs that do not code proteins, known as ncRNAs, which control diverse biological functions in the plant kingdom [5]. These ncRNAs are classified as small ncRNAs, comprising miRNAs (21–24 nt long) [6], small interfering RNAs (siRNAs) [7], Piwi-interacting RNAs (piRNAs) (generally found in animals) [8] and lncRNAs (>200 nt long) [9]. circRNA are another class of ncRNA generated from pre-mRNA splicing, featuring closed 3′ and 5′ ends covalently [10]. In addition to these ncRNAs, small nucleolar RNAs (snoRNAs), ribosomal RNAs (rRNAs), and transfer RNAs (tRNAs) known as housekeeping ncRNAs are also found in plant species [11]. The main classes of ncRNAs, illustrated in Figure 1, contribute to various plant development pathways and abiotic and biotic stresses by modulating the expression of associated genes [12,13,14,15,16,17,18]. In this review, we discuss the biogenesis of major plant ncRNAs and their interplay with corresponding target gene(s) controlling plant responses to biotic and abiotic stresses and with key developmental processes, including flowering, pod and seed development, nodulation, and nutrient acquisition in various legume crop species.

## 2. Types, Origin, and Function of Major Regulatory ncRNAs

Plant ncRNAs are ubiquitous and versatile repressors [6]. The major ncRNAs found in plants are broadly classified as small ncRNAs comprising miRNAs, siRNAs, long noncoding RNAs (lncRNAs), and circular noncoding RNAs (circRNAs) [6,7,19,20,21]. miRNAs are endogenous ncRNAs, about 20–24 nt long, abundant in both animal and plant kingdoms. They originated from miRNA genes through the transcription process by RNA pol II followed by processing of primary transcripts into mature miRNA catalysed by DICER-like (DCL) proteins [6,22,23]. Eventually, the mature miRNA is incorporated into the ARGONAUTE protein to assemble a miRNA-induced silencing complex (miRISC) [24]. Primarily, miRNAs function at the post-transcription level by base pairing with cognate mRNA, degrading or inhibiting mRNA translation [7,25,26]. Likewise, siRNAs (~22 nt long) originated from DCL-catalyzed processing of double-stranded RNA (dsRNA) precursors [7,24]. Primarily, siRNAs are classified as (1) trans-acting siRNAs (ta-siRNAs) generated from long noncoding single stranded RNAs, (2) natural antisense transcript-derived siRNAs (nat-siRNAs) derived from natural antisense RNAs, and (3) siRNAs belonging to repetitive DNA or transposons (see [27]). They play a central role in DNA methylation, chromatin modification, and repression of distinct mRNA targets by trans-acting siRNAs [28]. lncRNAs are > 200 nt noncoding RNAs found in animals and plants, located in the cytoplasm and nucleus [29,30]. The major classes of lncRNAs are long intergenic RNAs (lincRNAs), natural antitranscripts (NATs), and intronic ncRNAs (incRNAs) [31,32]. They are transcribed by RNA polymerase II or III and polymerase IV/V [33]. These lncRNAs can serve as precursors of miRNAs and siRNAs and act as endogenous target mimics (eTM) competing for various miRNAs [34]. Moreover, they participate in chromatin topology modification [35], alternative spicing [36], post-translational regulation [37], and protein relocalization [38]. Further detail on plant-lncRNA function is available elsewhere [39,40,41]. Circular RNA is a covalently closed, single-stranded RNA molecule generated by back-splicing events, categorized into exonic circRNAs, intronic circRNAs, intergenic circRNAs, and UTR circRNAs [10]. Our understanding on the role of circRNAs in plants is still limited [42].

## 3. Evolution, Conservation, Species Specificity, Tissue Specificity, and Genotype- and Stress-Dependent Expression of ncRNAs

Among the various plant ncRNAs, miRNAs are evolutionarily highly conserved in plant species ranging from nonvascular mosses to flowering monocots and dicots [43,44]. Researchers have found that individual plant species harbor conserved miRNAs and species-specific miRNAs [45]. Various conserved miRNAs have been reported, viz., miR156, miR159, miR165, and miR169 [44]. Likewise, species-specific miRNAs, viz., miR4414, miR5037, miR5208, miR5287, and miR5559, have been reported in *Astragalus chrysochlorus* [46] and *Ammopiptanthus mongolicus* [47] legume species and may be specifically expressed in legumes. De la Rosa (2020) [45] found that genes for miR398 are distributed throughout spermatophytes, but miR2119 was only found in legume species, indicating its recent emergence. The function of miR2119 in *Phaseolus vulgaris* and its presence in other legumes such as *Glycine max*, *Medicago truncatula*, and *Arachis hypogaea* have been reported [43,48]. Conserved miRNAs are involved in regulating common plant developmental processes, e.g., plant morphology; however, species-specific miRNAs may regulate special trait development, e.g., legume-specific cell processes and nodulation in legumes [49,50]. Expression patterns of conserved miRNAs vary greatly across plant species [51]. This has been supported by various research groups [52,53,54] by observing the abundance of miR398 expressed in the leaves but not the inflorescence of *Arabidopsis*. Conversely, *M. truncatula* had a high abundance of miR398 expressed in flowers but not in leaves [51]. Moreover, the expression of miRNAs varies from tissue to tissue, genotype to genotype, and stress to stress [44]. Under drought stress, Barrera-Figueroa et al. (2011) [55] noted 20 miRNAs differentially expressed in IT93K503-1 (drought-tolerant) and CB46 (drought-sensitive) cowpea genotypes. Among these, nine were only expressed in one genotype and not the other. Likewise, 11 miRNAs were expressed in one cowpea genotype but not in other genotypes under water stress, indicating genotype-dependent expression of miRNAs [55].

In groundnut, the leaves, flowers, and roots had higher expression of miR3 and miR7 than the seeds, and the stems’ leaves, roots, and stems had higher expression of miR156 than the flowers and seeds, suggesting tissue-specific expression of miRNAs in legumes [56]. Similarly, for lncRNAs, Das et al. (2019) [57] noted a higher expression of Cc_lncRNA-765 and its target mRNA, a carboxy peptidase-like mRNA, in seed tissue than pod tissue in pigeon pea. The reverse was true for Cc_lncRNA-2150 in pods compared with seeds at 30 days after podding. Similarly, Tridade et al. (2010) [58] reported upregulatory activity of miR398 and miR408 in response to drought stress in *M. truncatula*, but others reported downregulatory activity of miR398a, miR398b, and miR408 under salinity and alkalinity stress [59]. In the same way, miR399 was upregulated under phosphorus deficiency and downregulated under nitrogen deficiency in common bean [60]. Golicz et al. (2018) [61] witnessed sequence homology of four lncRNAs in various legume species, including soybean, chickpea, and *M. truncatula*. Several plant and legume plant-based ncRNA databases, viz., SoyKB [62], PNRD [63], PLNlncRbase [64], GreeNC [65], and PLncPRO [66], have been developed to discover and functionally annotate ncRNAs. The continual evolution of ncRNA databases and advances in computational and comparative analysis will improve our understanding of the conservation of ncRNA genes with their precise mode of function across various species in the plant kingdom [41].

## 4. ncRNAs Mediating Plant Immunity against Attacking Pathogens

Among the various biotic stresses, infections caused by fungi, bacteria, viruses, and nematodes significantly damage plants, resulting in substantial yield losses in various legumes [67,68,69]. Plants evoke a two-layer defence mechanism known as pathogen-associated molecular patterns (PAMPs)-triggered immunity (PTI) and effector-triggered immunity (ETI) against evading pathogens [70,71,72]. A series of protein-encoding gene(s), viz., pathogenesis-related genes, R genes, and other defense-related genes, are switched on and mediate conferring ETI and PTI in response to pathogen attack [72]. However, the emerging RNA-seq-based transcriptome sequencing approach underpinned a plethora of ncRNAs modulating various pathogenesis-related genes and R genes, thus regulating the plant immune response to various attacking pathogens [73]. ncRNAs play vital role in protecting plants from pathogen invasion by modulating ROS, the MAPK signalling cascade, and various TFs involved in switching on defence gene(s) [67,69]. Likewise, these, ncRNAs also participate in turning on downstream R genes and genes encoding pathogenesis-related proteins/phenolic compounds/phytoalexins and various phytohormone signalling in response to pathogen attack, thereby regulating plant disease resistance [67,69,74].

To establish the role of miRNAs regulating *Ascochyta* blight (AB) resistance in chickpea, Garg et al. (2019) [69] unveiled 651 miRNAs, including 173 novel miRNAs, in response to AB infection in contrasting parents. The authors noted both upregulation and downregulation of various miRNAs identified at various time points of AB infection. Functional analysis suggested the role of these miRNAs regulating AB resistance by evoking various TFs, phytohormones, and pathogenesis-related protein and R genes. Of the 12 miRNAs, 5 miRNAs, such as miR482b-3p, miR167c, miR171b, miR157a, and miR5232, were validated through degradome sequencing [69] (see Table 1). The predicted target genes of the above corresponding miRNAs were identified as *Ca_08122* (encoding CC-NBS-LRR), *Ca_19433* encoding (Dof zinc finger protein), *Ca_00359* (encoding ERF), *Ca_15107* (encoding senescence-associated protein), and *Ca_12185* (encoding calcium-transporting ATPase). The study also explained the possible causative mechanism of AB infection in the susceptible genotype through the upregulation of miR482b-3p, miR159k-3p, nov_miR66, and miR171 miRNAs and the downregulation of the corresponding target genes encoding NBS-LRR, PR protein, a serine-threonine kinase, and PPR proteins, allowing AB infection [69] (see Table 2). Considering *fusarium* wilt (FW) in chickpea, Kohli et al. (2014) [68] reported 122 conserved and 59 novel miRNAs by sequencing small RNA from ICC4958, a FW-tolerant chickpea genotype. The authors noted the upregulation of FW-responsive miRNAs, viz., miR530 (targeting zinc knuckle proteins) and the microtubule-associated proteins miR156_1 miR156_10, car-miR2118, and car-miR5213 (targeting TIR-NBS-LRR). Deep sequencing of two soybean cultivars, Hairbin xiaoheidou (resistant to soybean cyst nematode) and Liaodou 10 (susceptible to soybean cyst nematode), unearthed 364 and 21 novel miRNAs [74]. Among the conserved miRNAs identified, MiR169 was upregulated in Liaodou 10 and downregulated in Hairbin xiaoheidou; however, MiR319 (targeting *TCP* gene) was upregulated in both cultivars.

Likewise, gma-miR390b was upregulated by soybean cyst nematode (SCN) in Hairbin xiaoheidou and downregulated in Liaodou 10. Of the 21 novel miRNAs identified, soy_1, soy_2, and soy_3 (targeting HD-ZIP transcript factor) and soy_9 (targeting calmodulin) were noted [74]. Likewise, 60 SCN-responsive miRNAs were identified in KS4607 (susceptible) and KS4313N (resistant) soybean genotypes using deep sequencing and miRDeep2 pipeline analysis [74]. Among the SCN-responsive miRNAs, various conserved miRNAs, viz., miR171, mir399, miR159, and miR398, and legume-specific miRNAs, viz., miR9750, miR2119, and miR1512, were identified. Of the DE miRNAs, 34 miRNAs were upregulated; notably, miR159b-3p, miR159f-3p, and miR972 were downregulated in the susceptible cultivar, while 14 miRNAs were upregulated and miR2119, miR398a, and miR398b were downregulated in the resistant cultivar [67]. In groundnut, small RNA transcriptome sequencing of pod rot infected groundnut using Illumina HiSeq 2000 elucidated 334 miRNAs, of which 97 were downregulated and 27 were upregulated [104]. Functional validation of selected miRNA, viz., ahy-miR396e-5, was downregulated, but its target gene, *c39419_g1_i1*, was upregulated after infection. Likewise, ahy-miR3509-5p, ahy-miR166f, and ahy-miR159b were downregulated after infection, but their corresponding target genes, *c40055_g1_i3*, *c31393_g1_i1*, and *c41016_g4_i1*, were upregulated [104]. However, a complete understanding of ncRNAs identified as regulating disease resistance in legumes remains elusive. Future identification of novel disease-responsive ncRNAs will provide novel insights into the interplay of ncRNAs and the plant immune response for developing disease-resistant legumes.

## 5. Deciphering the Molecular Mechanisms of ncRNAs Regulating the Response of Legumes to Water Stress

Drought stress is the most important abiotic stress globally, affecting all plant growth and developmental stages, and ultimately reducing crop yields [145]. Plants adapt to a water deficit environment by evoking various physiological, biochemical, metabolic, and molecular mechanisms [146]. Many QTL/genes contributing to drought tolerance have been investigated in various legumes [147]. Indeed, the participatory role of various regulatory ncRNAs and their corresponding target gene(s) controlling drought stress have been deciphered in various plant species, including legumes [55,95,113,125]. A plethora of novel drought-responsive miRNAs have been identified in legume crops—157 in cowpea [55], 143 and 128 in grass pea [121], and 284 in chickpea [92]—and 3457 high-confidence lncRNAs have been identified in chickpea [66]. ncRNAs confer drought tolerance by regulating gene(s) encoding various regulatory TFs and osmoregulatory/osmoprotective compounds by activating hormone signalling and antioxidants that minimize oxidative stress/reactive oxygen species (ROS) activity in plants under water stress [55,113,121].

Deep sequencing of two contrasting cowpea genotypes —CB46 (drought-sensitive) and IT93K503-1 (drought-tolerant)—grown under normal and drought stress conditions enabled in identifying 44 drought-responsive miRNAs (30 upregulated and 14 downregulated) [55] (see Table 1). Notably, miR156 (targeting SPB transcription factors) was upregulated and miR169 (targeting NFYA5) was downregulated in both genotypes under water stress. miR160a and miR160b (targeting *Auxin Response Factors*) and vun_cand015 (targeting bHLH transcription factor) were upregulated in the tolerant cultivar, and miR2111 (targeting *Kelch repeat-containing F-box proteins*) was upregulated in the drought-sensitive cultivar [55].

To predict the possible role of miRNAs in producing osmoprotective compounds to regulate the drought stress response, Shui et al. (2013) [113] elucidated and validated the active role of vun-miR5021, vun-miR156b-3p and vun-miR5021 (targeting *CPRD86*), vun-miR156b (targeting 1-pyrroline-5-carboxylate synthase *P5CS* involved in proline synthesis), and vun-miR156f (targeting *multicystatin* gene encoding cystatins) miRNAs in leaf and root tissue of two contrasting cowpea genotypes (Danlla and Tvu7778) under water stress.

A study on the participatory role of conserved miRNAs—miR398a/b and miR408—in regulating water stress in pea revealed the downregulation of these miRNAs in root and shoot tissue under water deficit conditions [125]. However, the copper superoxide dismutase, *CSD1* target gene of miR398a/b, was upregulated, suggesting an inverse relationship between the target gene and the involved miRNA controlling water stress in pea. Similarly, De la Rosa et al. (2019) [48] supported the upregulatory role of *CSD1* and *ADH1* mRNAs targeted by miR398 and miR2119 in common bean adapting to drought stress.

Grasspea sequencing of small RNA identified numerous drought-responsive miRNAs [121]. Among the differentially expressed miRNAs, lsa-miR-169b, lsa-miR-319, lsa-miR-398, lsa-miR786, lsa-miR1361, and lsa-mir-156 were upregulated, and lsa-miR897, lsa-miR969, lsa-miR186, and lsa-mir-1520b were downregulated. lsa-miR-319 and lsa-miR-398 were predicted to target the *TCP* gene and cytosolic *CSOD1* and chloroplastic *CSOD2* genes, respectively [121]. In chickpea, small RNA sequencing of root tissues under water stress identified 284 miRNAs [95]. Functional validation of selected miRNAs, including miR397, miR398, miR164, and miR399 targeting *LACCASE4*, *COPPER SUPEROXIDE DISMUTASE*, *NAC1*, and the *PHO2/UBC24* gene, respectively, showed an inverse relationship under drought stress [95]. Illustrating the role of abiotic stress responsive lncRNA, Singh et al. (2017) [66] identified a total of 3457 high-confidence lncRNAs responding to drought and salinity stress in chickpea. The drought sensitive genotype ICC1882 showed the least number of 126 differentially expressed lncRNAs at the early reproductive stage, while a large number of lncRNAs exhibited downregulation under drought stress in all the tested samples. In parallel, a large number of lncRNA showed differential expression at the early reproductive stage in the ICC4958 drought tolerant chickpea genotype [66].

Considering the role of circRNAs attributing drought tolerance, Dasmandal et al. (2020) [148] uncovered numerous drought responsive differentially expressed circRNAs in chickpea and soybean. The authors also predicted three eTMs those acted as sponge for miRNAs that target *Glyma.18G065200.1* gene in soybean, and *XM_004517122*, *XM_027336693* genes in chickpea. The functional role of these targeted genes was associated with hormone signalling and various transcription factors under drought stress [148]. Further mechanistic understanding of ncRNAs and the corresponding target gene(s) will enhance our understanding of ncRNAs regulating drought tolerance in legume crops.

## 6. Role of ncRNAs in Plant Adaptation to Salinity Stress

The rapid conversion of uncultivable land to cultivated land and the excessive use of irrigation water have increased salinity, which is a major challenge for crop growth, including legumes, and causes significant yield losses [149]. Plants orchestrate various biochemical and molecular mechanisms to survive the increasing salinity stress [149], including ncRNAs [15,82,83,102], which target genes related to photosynthesis, TFs regulating growth, genes related to salinity-responsive hormone signalling, genes that minimize the uptake of toxic ions, viz., Na^+^, and genes that limit ROS activity [15,83,95].

Paul et al. (2011) [114] investigated the role of miRNAs controlling salinity stress in cowpea and recovered 18 conserved miRNAs (e.g., miR160, miR156/157, miR159, miR169, miR172, miR408) from root tissue and identified 15 corresponding target gene(s) as TFs (e.g., *ARF*, *SBP*, *AP2*, *TCP*). Functional validation through quantitative real-time PCR (qRT-PCR) revealed the upregulation of seven miRNAs under salinity stress.

Transcriptome analysis of root apex treated with salinity stress using miRDeep2 identified 66 salt-responsive miRNAs in soybean, of which 14 were upregulated (notably, miR172f and miR390e) and 22 were downregulated (notably, miR399a/b, miR1512b, miR156g, and miR156j) under salinity stress [80]. The predicted putative target genes of miR399a/b were *Glyma.14G188000*, *Glyma.15G074200*, *Glyma.08G359400*, and *Glyma18G177400* (encoding multicopper oxidases) and *Glyma03G021900* (encoding a growth-regulating factor). Likewise, *Glyma.02G281100* and *Glyma.14G033500* encoding LRR receptor-like kinases were the target genes of miR390e [83]. Subsequently, strand-specific transcriptome sequencing identified 3030 lincRNAs and 275 lncNATs in soybean roots under salinity stress [82]. Importantly, 75% of these lncRNAs were upregulated under salinity stress. Genome-wide scanning of salinity-responsive miRNAs elucidated 876 miRNAs related to salinity and alkalinity stress in *M. truncatula* [59]. Thirty-five miRNAs (including mtr-miR156 family, mtr-miR159a, and mtr-miR171) were upregulated under salinity and alkalinity stress, and eight miRNAs (including mtr-miR171e-3p, mtr-miR2628, mtr-miR398a-3p, mtr-miR398a-5p, and four novel miRNAs) were downregulated under both stresses [59]. Functional validation of miR319 (targeting *MTR_3g011610*, *MTR_1g102550*, and *MTR_1g052470*) and miR408 (targeting BBLP and *MTR_8g089110*) indicated their participatory role in salinity and alkalinity stress tolerance [59]. In chickpea, small RNA sequencing of root tissues treated with salinity stress identified 284 miRNAs [95]. Inverse correlation patterns of miRNA397, Car-novmiR2, and Car-miR5507 targeting the *LACCASE4*, *HAK5*, and *CIPK23* genes, respectively, were observed at the transcript level regulating salinity stress tolerance in chickpea [95].

A genome-wide survey of lncRNA through transcriptome analysis in groundnut identified 1442 lncRNAs [102]; notably, TCONS_ 00292946 lncRNA was downregulated in roots within 12 h of salinity stress but upregulated at 24 h. Expression of TCONS_00176941 was upregulated within 12 h in roots and downregulated within 12 h of salinity stress in leaves, while TCONS_00011551 was upregulated under salinity stress [102]. Wang et al. (2015) [15] investigated the role of lncRNAs involved in regulating the salinity stress response and conferring tolerance by alleviating ROS-related stress in *Medicago truncatula*. The authors identified the functional role of various lncRNAs attributing to salinity tolerance, including TCONS_00116877, which induced the *Medtr7g094600* gene encoding glutathione peroxidase to minimize ROS-derived stress in roots (see Table 2).

Alzahrani et al. (2019) [116] uncovered 1220 salt-responsive miRNAs by small RNA sequencing of two contrasting faba bean (*Vicia faba*) genotypes for salinity stress response (ILB4347 tolerant and Hassawi-3 sensitive). The Hassawi-3 genotype had 284 upregulated and 243 downregulated miRNAs, while ILB4347 had 298 upregulated and 395 downregulated miRNAs in the control and under salinity stress. The target gene(s) were predicted to encode TFs, laccases, superoxide dismutase, plantacyanin, and F-box proteins in addition to genes involved in hormone signal transduction, phosphatidylinositol signalling, and the MAPK signalling pathway [116].

## 7. Contribution of ncRNAs Attributing Plant Adaptation under Metal Toxicity Stress

Metal toxicity is an abiotic stress increasingly faced by plants due to rapid industrialization, excessive use of inorganic fertilizers, and overuse of irrigation water contaminated with heavy metals, especially cadmium and mercury [150]. Among the various complex molecular mechanisms, identifying the role of ncRNAs, including miRNAs and lncRNAs, is a potential approach for minimizing metal toxicity in plants [77,151].

**Figure 2:**
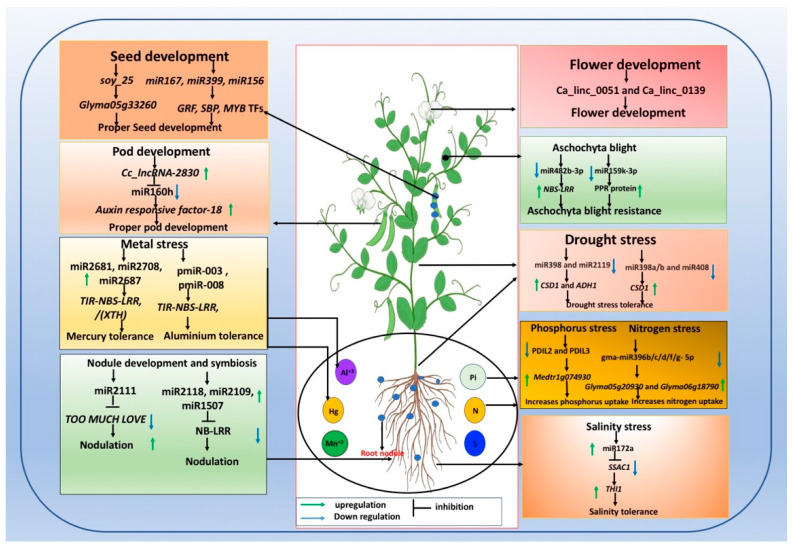
ncRNA module controlling various abiotic and biotic responses and developmental pathways in legume plants. Increased expression of Cc_lncRNA-2830 sequesters miR160h, resulting in upregulation of *Auxin responsive factor-18* allowing proper pod development [57]. The role of Soy_25 miRNA targeting *Glyma05g33260* gene attributing seed development is noteworthy [87] in soybean. In response to aschochyta blight attack, downregulation of miR482b-3p and miR159k-3p enhance expression of NBS-LRR and PR, respectively, inhibiting pathogen attack [69]. Under water stress, the downregulatory activity of miR398 and miR2119 increases the expression of *CSD1* and *ADH1* genes contributing to drought tolerance [48]. Under excess salinity stress, induction of miR172a cleaves mRNA transcripts of salt-suppressed AP2 domain-containing genes, allowing high expression of thiamine biosynthesis gene *THI1* that ultimately enables transcription of the salinity tolerance regulator in soybean [126]. For nutrient deficiency stress, such as phosphate, downregulation of PDIL2 and PDIL3 lncRNAs increases the expression of *Medtr1g074930* and phosphate uptake [17]. The repressive action of gma-miR396b/c/d/f/g-5p upregulates *Glyma05g20930* and *Glyma06g18790* genes, increasing N uptake [86]. During mercury metal stress, induction of miR2681, miR2708, and miR2687 enhances expression of the *TIR-NBS-LRR/*(*XTH*) gene imparting resistance against mercury [77]. During nodulation and symbiosis, miR2111 inhibits expression of the *TOO MUCH LOVE* gene, upregulating the nodule development process [130], while upregulation of miR2118, miR2109, and miR1507 enables nodulation by repressing *NB-LRR* genes [142].

Deep sequencing and high-throughput degradome analysis of heavy metal mercury-treated and mercury-free *M. truncatula* seedlings identified 201 miRNAs [77]. Of these, 12 were specifically induced under mercury stress. Functional analysis of miR2681, miR2708, and miR2687 targeting the *TIR-NBS-LRR* (encoding disease resistance protein), *TC114805* (encoding salinity tolerance protein), and *XTH* gene coding xyloglucan endotransglucosylase/hydrolase contributing to cell wall development, respectively, was deciphered (see Figure 2). Thus, these miRNAs and the putative target could be an important approach for regulating heavy metal stress tolerance in *M. truncatula* [77]. Earlier, Zhou et al. (2008) [152] reported the upregulatory role of miR171, miR319, miR393, and miR519 and the downregulatory role of miR166 and miR398 in response to Al^3+^ treatment in *M. truncatula*. Subsequently, Chen et al. (2012) [78] elucidated 326 known miRNAs and 21 new miRNAs responsive to aluminium toxicity using small RNA sequencing of Al^3+^-treated and Al^3+^-untreated *M. truncatula*. Functional characterization of selected miRNAs, viz., pmiR-003 and pmiR-008 (targeting genes encoding TIR-NBS-LRR resistance protein), revealed their possible role in mediating aluminium toxicity tolerance [78]. Twenty-eight miRNAs responsive to aluminium toxicity were recovered from roots and nodules in common bean using the miRNA-macroarray hybridization technique [99]. Functional validation of selected miRNAs revealed upregulation of miR164 targeting (*NAC1* TF), miR170 targeting (*SCL* TF), and miR393 targeting *TIR1*, and downregulation of miR157 targeting (*SPL*) and miR398 targeting (*CSD1*) under aluminium stress in common bean nodules [99]. Eleven miRNAs, viz., miR157, miR156, miR170, miR172, and miR319, exhibiting strong upregulation in root nodules, and 11 miRNAs, *viz.,* miR160, miR397, miR399, miR408, pvu-miR1509, and pvu-miR1514a, exhibiting strong downregulation in leaves or roots, were discovered under manganese toxicity in common bean [60] (see Table 2). Few toxic metal-responsive miRNAs have been reported in legumes. Therefore, further study is needed to gain insight into toxic metal-responsive miRNAs and their target genes and precise function.

## 8. Molecular Mechanisms of ncRNAs Regulating Nutrient Acquisition and Homeostasis in Legumes

Plants acquire essential nutrients by recruiting various physiological and molecular mechanisms via roots and soil for proper growth and development [153,154]. Of these mechanisms, the critical role of ncRNAs regulating the uptake of various macro- and micronutrients has been recognized [155,156].

Nitrogen (N)—serving as the source of various essential amino acids and acting as an important element for entire nitrogen metabolism—is a critical determinant for plant growth and development [157]. Emerging functional genomics approaches, viz., RNA-seq, can underpin the plethora of nitrate transporter QTLs, gene(s), and ncRNAs controlling N use efficiency (NUE) in plants [158]. However, the complete molecular mechanism of NUE/N homeostasis remains unclear in plants, including legumes.

Evidence for the miRNAs controlling the nitrogen response in plants has been reported [86,159]. The upregulation of pri-miR156 and pri-miR447c and downregulation of pri-miR169 and pri-miR398a were reported in *Arabidopsis* under nitrogen-limited conditions [160]. Several nitrogen-responsive miRNAs, viz., miR164, miR169, miR172, and miR397 in maize shoots and miR160, miR167, miR168, and miR169 in maize roots, under nitrogen deficiency conditions have been reported [159]. Likewise, several nitrogen-responsive miRNAs have been reported in legume crops [86]. Wang et al. (2013) [86] recovered 168 nitrogen-responsive miRNAs from small RNA sequencing of a low N tolerant (No.116 genotype) and low N sensitive (No.84-70 genotype) soybean genotype. The study revealed downregulation of gma-miR2606a/b-3p in the roots of variety No.116 and upregulation of gma-miR1512a-5p in the roots of variety No.84-70 under short-term low N. However, gma-miR396b/c/d/f/g-5p was downregulated in the shoots of No.116 and upregulated in the shoots of No.84-70 under short-term low N stress [86]. Moreover, some of the predicted miRNA targeting genes were predicted to play a role in protein degradation, viz., gma-miR156b/6f-5p (targeting *Glyma07g31580*) and gma-miR396bg-5p (targeting *Glyma05g20930* and *Glyma06g18790*), encoding E3 ubiquitin ligase and Cathepsin L1 (see Table 2).

Phosphorus (P) is the second most essential macronutrient required for basic biochemical and metabolic processes in plants, including legumes [161]. Plants usually uptake P in the form of inorganic phosphate (Pi). Thus, P deficiency limits overall plant growth and development. The involvement of several P-responsive ncRNAs has been elucidated in various plant species [14,17,160,162,163,164]. Likewise, previously P-responsive miRNAs have been reported in common bean [60,144], white lupin [119], soybean [165], *M. truncatula* [132], alfalfa [166,167], and lupin (*Lupinus albus*) [119]. Several conserved regulatory miRNAs, such as miR399 [162,168,169,170] and miR156, miR169, and miR2111 [160] regulating Pi homeostasis have been reported in *Arabidopsis*. Li et al. (2018) [13] confirmed the inductive role of miR399 (targeting phosphate transporter genes) and miR398 (targeting *Copper chaperone for SOD*) under low Pi stress in roots of *Medicago sativa.* However, the authors noted downregulation of miR156 (targeting *SPL* TF), miR159 (targeting *MYB* TF), miR160 (targeting *auxin response factor* TF), miR171 (targeting *GRAS* TF), and miR2643 (targeting *MATE*). The molecular mechanism involving IPS1 lncRNA serving as eTM for miRNA399 targeting *PHO2* gene expression and controlling Pi homeostasis has been established in *Arabidopsis* [34]. Under low Pi conditions, upregulation of the *PHR1* gene and miRNA399 inhibiting the *PHO2* gene (encoding transcript causing Pi transporter degradation) enabled high Pi acquisition in *Medicago sativa* [17]. Downregulation of PDIL2 and PDIL3 lncRNAs enhanced transcript expression of *Medtr1g074930*, a Pi transporter gene, enabling high Pi uptake under low Pi conditions. However, PDIL1 lncRNA serves as a target mimicry for miR399, inhibiting the degradation of *MtPHO2* transcripts that could downregulate the Pi transport gene and Pi uptake [17] (see Figure 2). To gain insight into the role of P-responsive circRNAs, Lv et al. (2020) [14] uncovered 120 differentially expressed cicrRNAs by transcriptome sequencing of two contrasting P-responsive soybean genotypes at different P levels. Gene ontology (GO) enrichment analysis predicted that the putative role of the differentially expressed circRNAs is related to nucleoside binding, organic substance catabolic processes, and oxidoreductase activity [14]. Low P-responsive circRNAs could be targeted for improving phosphorus use efficiency in soybean. Thus, a complex network of ncRNAs and their corresponding target gene(s) play a central role in regulating Pi homeostasis in plants.

## 9. Regulatory Role of ncRNAs for Shaping Developmental Processes in Legume Species

Apart from various biotic and abiotic stresses, ncRNAs, including miRNAs (conserved and nonconserved) and lncRNAs, play a pivotal role in regulating plant growth and development and in various metabolic pathways, which has been investigated in various legume species [61,91,92,96,103,120,171,172]. Small, deep RNA sequencing analysis of seven chickpea tissues was used to investigate a comprehensive set of 440 known and conserved and 178 novel miRNAs targeting various TFs and gene(s) that control various developmental processes, including leaf, flower, pod, and root development and various metabolic processes in chickpea [92] (see Table 1). Subsequently, small RNA sequencing of chickpea leaves and flowers discovered 157 conserved and novel miRNAs that regulate various developmental processes and stress responses [12]. Of the identified miRNAs, miR156, miR159, miR160, miR162, miR164, miR172, miR408, and miR393 targeting *SBP*, *MYB*, *ARF*, *DCL1*, *HD-zip*, *AP2*, *F-box protein*, and *plantacyanin* encoding genes, respectively, contribute to various plant development processes [12] (see Table 2). The authors also disclosed the role of *TAS3*-derived tasiRNA targeting *ARF2*, *ARF3*, and *ARF4* transcription factors controlling auxin response, and thus contributing to development pathways in chickpea. In this context, Jagadeeswaran et al. (2009) [51] identified and characterized Tas3-siRNAs from *M.trucatula* and also functionally validated three *ARF* genes targeted by these Tas3-siRNAs.

Considering ta-siRNA participating in regulating compound leaf and flower development in *L. japonicus*, Yan et al. (2010) [173] established the role of *Reduced leaflet1* (*REL1*) and *Reduced leaflet3* (*REL3*) genes encoding homologs of Arabidopsis (*Arabidopsis thaliana*) ‘SUPPRESSOR OF GENE SILENCING3′ and ‘ARGONAUTE7/ZIPPY’, respectively, key components required for ta-siRNA biogenesis. Positional cloning analysis of *REL1* and *REL3* genes revealed that the ta-siRNA pathway critically plays significant role in controlling compound leaf and flower development in *L. japonicus* [173]. Likewise, elucidating the role of *trans-acting siRNA3* (*TAS3*) involved in leaf margin indentation and organ separation, Zhou et al. (2013) [174] examined that Mt-AGO7/LOBED LEAFLET1 is required for the biogenesis of ta-siRNA to suppress the expression of *Auxin Response Factors*. Evidence of lobed leaf margin and widely spaced lateral organ phenotype demonstrated in the *ago7* mutant suggested that *TAS3* plays a negative role in leaf margin and lateral organ development in *M. truncatula* [174]. Examining the functional role of lncRNA associated with flower development, Khemka et al. (2016) [91] discovered a total of 2248 long intergenic noncoding RNA obtained from the results of RNA-seq data of eight flower development tissues. Further, qRT-PCR result showed specific expression of Ca_linc_0051 and Ca_linc_0139 lncRNA in the flower bud and shoot apical meristem stage, confirming their possible role in flower development in chickpea [91].

Glazińska et al. (2019) [120] reported several miRNAs regulating floral development, viz., Ll-miR280, Ll-miR281, and Ll-miR285 (possibly targeting *ARF6* and *ARF8*); Ll-miR445 and Ll-miR130 (targeting *TCP4* and *MYB33*); and Ll-miR329/miR160-5p, Ll-miR332/miR160-5p, and Ll-miR333/miR160-5p miRNAs regulating flower abscission in yellow lupin (*Lupinus luteus* L.). Among the siRNAs identified from this study, Ll-siR173, Ll-siR4, and Ll-siR13 exhibited upregulation and downregulation of Ll-siR208, suggesting the active role of siRNA functioning in lupin pedicel [120]. Das et al. (2019) [57] explored a plethora of lncRNAs and target miRNAs forming an endogenous target mimicry leading to pod and seed development using transcriptome analysis of tissue collected during anthesis and pod development in pigeon pea. Functional validation revealed that sequestering Cc-miR160h by Cc_lncRNA-2830 enabled the transcription of *XM_020377020* (encoding auxin response factor 18-like protein) during pod development at 10 and 20 days after anthesis (DAS). However, expression of Cc_lncRNA-2830 at 30 DAS decreased, which upregulated Cc-miR160h and degraded the *XM_020377020* transcript [57] (see Figure 2).

To better understand the role of miRNAs regulating embryo and pod development in groundnut, small RNA profiling and degradome sequencing identified 70 known and 24 novel miRNA families [105]. Functional validation of selected miRNA, viz., miR164, miR167, miR172, miR390, miR7502, and miR9666, using qRT-PCR revealed upregulatory activity; however, miR156, miR396, miR894, miR1088, miR4414, and miRn8 were significantly downregulated during early embryo and pod development [105]. In groundnut [Chen et al. (2019) [109], 29 known and 132 novel miRNAs were identified when exploring the participatory role of miRNAs in embryo development under calcium deficiency. Transcriptome analysis identified 52 differentially expressed genes targeted by 20 miRNAs. Functional validation of selected miRNAs, viz., ahy_novel_miRn129 and ahy_novel_miRn130 (targeting transcription factor “LONE- SOME HIGHWAY” (LHW) encoding bHLH transcription factor), exhibited upregulation under calcium deficiency [109]. The same study showed upregulation of ahy_novel_miRn112 and downregulation of target gene (*NAM*/*CUC*), while ahy_novel_miRn23 (targeting *CYP707A1* and *CYP707A3* encoding ABA 8′-hydroxylase) was significantly upregulated, and ahy_novel_ miRn30, ahy_novel_miRn29, and ahy_novel_miRn38 with their corresponding targets *TEOSINTE BRANCHED1*, *CYCLOIDEA*, and *PROLIFERATING CELL FACTORS 4* (*TCP4*) involved in jasmonic acid biosynthesis were downregulated [109]. Thus, these miRNAs with their target gene(s) modulate embryo development in groundnut.

As the entire underlying molecular mechanism for seed development, from embryogenesis and filling to maturation, remains elusive [98], several investigations have reported the involvement of various ncRNAs regulating seed development in grain legumes [92,93,98,102]. To investigate the contributory role of ncRNA involved in the seed development process, transcriptome sequencing of seed samples using an Illumina Genome Analyzer IIx uncovered 72 known and 39 new miRNAs involved in seed development, particularly embryogenesis, dormancy, and maturation, in common bean [98]. The notable miRNAs and the target genes involved in regulating seed development were MIR156 repressing *SPL*; MIR169 repressing *NF-YA1* and *NF-YA9*; MIR399 inhibiting *SUT1* related to sucrose transport; MIR399 inhibiting *PHO2* contributing in phosphorus allocation; MIR160 repressing *ARF10*, *ARF16*, and *ARF17*; MIR167 inhibiting *NCED1* associated with ABA synthesis; and MIR395 repressing *SULTR2;1*, *APS* contributing to sulphate assimilation and allocation during seed filling [98]. Likewise, genome-wide profiling of miRNAs using small RNA sequencing of seeds of two contrasting chickpea genotypes—Himchana1 (low seed weight) and JGK3 (high seed weight)—unfolded 113 known and 243 novel miRNAs controlling seed development in chickpea [93] (see Table 1). The target genes of identified miRNAs contributing to seed development were predicted to be *SPL*, *GRF*, *MYB*, *ARF*, *HAIKU1*, *SHB1*, *KLUH/CYP78A5*, and *E2Fb.* Low expression of Car-miR319 and Car-miR166 and upregulation of their corresponding target genes, bZIP and homeobox-*REVOLUTA* TFs, in JGK3 indicated their important role in seed size determination in chickpea [93]. The authors also located 19 miRNAs and 41 target genes in previously identified QTLs contributing to seed size.

The role of various conserved miRNAs, viz., miR167, miR390, miR164, miR399, miR156/157, miR1511, and mir319, and seven novel miRNAs, viz., NovmiR13, NovmiR12, and NovmiR04, regulating seed development in narrow-leafed lupin was confirmed in studies by DeBoer et al. (2019) [118]. Differential expression analysis revealed upregulation of Lan-miR-156a-2, Lan-miR-164-3, Lan-miR-167a/c, Lan-miR-319, Lan-miR-399b/c, NovmiR12, and Nov-miR13 in seeds, indicating their role in regulating seed development in lupin [118]. The role of miRNAs controlling genes related to sugar metabolism during seed development is worth mentioning [87,175]. In soybean, deep sequencing and degradome sequencing of developing soybean seed revealed several miRNAs targeting genes that contribute to seed development [87]. Among the identified miRNAs, functional validation of gma-miR1530 revealed its role in inhibiting the target transketolase gene that contributes to switching carbon assimilation to energy metabolism during seed development. Likewise, the pentatricopeptide repeat protein-encoding gene was targeted by Soy_3 and Soy_16, while Soy_25 (targeting *Glyma05g33260* homolog of Arabidopsis “SUPPRESSOR OF GENE SILENCING 3”) contributing to seed development was identified [87] (see Figure 2). A total of 484 miRNAs were recovered from small RNA sequencing of four contrasting soybean lines with high protein/high oil, high protein/low oil, high oil/low protein, and low protein/low oil [175]. Functional validation of selected miRNAs, including *Glyma.13G035200* and *Glyma.14G156400* (encoding alcohol dehydrogenase 1) targeted by Gma-miR2119, *Glyma.04G178400* (encoding ADP-glucose pyrophosphorylase family protein) targeted by Gma-miR1521a, and *Glyma.19G094000* (related to sugar synthesis and metabolism) targeted by miR156, using RT-qPCR indicated their significant role in controlling storage genes during seed development in soybean [175].

Computational analysis identified 347 candidate circRNAs in groundnut [110]; the differential expression of 29 circRNAs was upregulated in seeds collected from RIL 8107′ at 35 days after flowering (DAF) and RIL 8106′ at 35 DAF, confirming their contributory role in seed development [110]. Likewise, Ma et al. (2020) [111] detected 9388 known and 4037 novel lncRNAs in groundnut, of which 1437 lncRNAs were differentially expressed. Functional validation of selected lncRNAs confirmed their role in seed development. The participatory role of miR156, miR159, miR171, and miR14 (targeting genes related to cellular amino acid metabolism, fatty acid metabolism, and lipid metabolism) in groundnut is noteworthy [56].

To establish the role of the DCL2-dependent 22-nucleotide siRNA (derived from long inverted repeats) regulating *chalcone synthase* (*CHS*) genes attributing seed coat colour in soybean, a study conducted by Jia et al. (2020) [176] revealed that CRISPR/Cas9-driven loss-of-function mutants of *DCL2* (*GmDCL2a* and *GmDCL2b*) caused changes in seed coat colour from yellow to brown in *Gmdcl2a/2b* mutants in soybean. Thus, this study confirmed that DCL2 controls soybean seed coat colour via generating siRNA from long inverted repeats [176].

Further identification of ncRNAs related to the development process, especially pod and seed development, and their precise function will provide better new avenues for improving pod and seed size and thus grain yield in legumes.

## 10. ncRNAs Orchestrating Nodulation, Symbiosis, and Root Development Processes

Legumes are unique due to their inherent ability of forming root nodules in association with active soil rhizobacteria that assist in fixing atmospheric nitrogen [1]. The underlying molecular mechanism and around 200 genes involved in fixing atmospheric nitrogen in soil through nodulation and symbiosis have been deciphered [177,178]. Likewise, evidence of small RNAs, including miRNAs involved in nodule development and root symbiosis, has been reported in various model legumes, viz., *M. truncatula*, *L. japonicus*, and soybean [49,51,76,133,179,180,181,182,183]. The greater abundance of miR172 in root nodules than leaf tissue in *Medicago truncatula* [76], *Lotus japonicus* [138], common bean [60], and soybean [134] suggests its active role in nodulation. The role of MIR166 (targeting *HD-ZIP III* TF genes contributing to root nodule development) in *Medicago truncatula* was revealed by its overexpression, which downregulated *HD-ZIP III*, inhibiting symbiotic nodules and lateral root development [132]. Similarly, in soybean, miR166 and miR396 (targeting *HD-ZIP III* TF and *cysteine protease* gene, respectively) depicted downregulation during nodulation in soybean [49].

Considering the potential role of miRNAs involved in signalling pathways related to nodule infection and N_2_ fixation, De Luis et al. (2012) [138] demonstrated that the induction of miR171c in root nodules targeting *NSP2* TF is correlated with bacterial nodule infection. While the induction of miR397 is noted strictly in rhizobial bacteria-infected active N_2_ fixing nodules, it participates in contributing to nitrogen fixation-related copper homeostasis and also targets the laccase copper protein family gene in *Lotus japonicus* [138]. Subsequently, the negative role of gma-miR171o and gma-miR171q miRNAs regulating soybean nodulation was functionally validated [184]. The authors demonstrated that the regulatory expression of two TF genes, *GmSCL-6* and *GmNSP2* (target genes of gma-miR171o and gma-miR171q miRNAs), plays an active role in the expression of *NIN*, *ENOD40*, and *ERN* genes involved in the nodulation process in soybean. Among the other miRNAs attributed to the nodulation process, the regulatory circuit of nodule development controlled by miRNA172-targeting *AP2* and miRNA156-regulating miRNA172 expression in soybean has been investigated [49,134].

**Figure 3:**
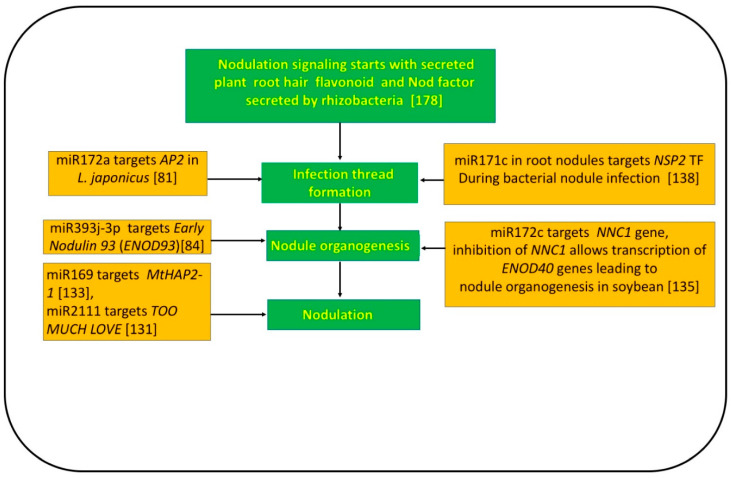
Role of selected miRNAs regulating nodulation process in legume plant [81,84,131,133,135,138,178].

Various research groups [140,185,186] have suggested that the negative regulation of miR171h targeting *MtNSP2* is needed for nodule formation and the mycorrhizal signalling pathway in *Medicago truncatula*. Overexpression of miR396b in roots of *Medicago truncatula* impaired root growth and diminished mycorrhizal colonization by targeting six growth-regulating factor genes (*MtGRF*) and two *bHLH79*-like genes, indicating the significant role of miR396b in root growth and mycorrhizal colonization [139] (see Table 2). Further insights into the underlying complete molecular mechanism of miR172c controlling rhizobial infection and precise nodulation regulation were elucidated in soybean [135]. The authors postulated that the absence of rhizobia *Nodule Number Control1* (*NNC1*) suppresses the transcription of *ENOD40* genes in soybean. However, in the presence of rhizobia, nod factor receptors induced a signal cascade that evokes the upregulation of miR172c targeting the *NNC1* gene. Thus, the inhibition of *NNC1* allows transcription of *ENOD40* genes leading to nodule organogenesis in soybean (Figure 3).

Likewise, considering the underlying role of miR172a in rhizobial infection during symbiosis, Holt et al. (2015) [81] supported that the inductive activity of miR172a in *L. japonicus* roots requires the presence of both active rhizobial bacteria and bacterial Nod factor signalling during the early stage of symbiotic infection. Possible targets of miR172a were predicted to be the *RAP2-7-like1*, *AP2-like1*, and *AP2-like2* genes during bacterial symbiosis. Subsequently, Yan et al. (2015) [84] functionally demonstrated that the overexpression of miR393j-3p miRNA targeting a nodulin gene *Early Nodulin 93* (*ENOD93*) significantly inhibited nodule formation in soybean. Turner et al. (2012) [85] monitored the high expression of *Glyma10g10240* and *Glyma17g05920* (targets of miRNA169), which encode HAP proteins that contribute to nodule development.

The role of miR169 in regulating nodule development (transition from meristematic to differentiated cells) in *M. truncatula* by targeting the *MtHAP2-1* novel symbiosis-specific TF gene has been established [133] (Figure 3). Li et al. (2010) [129] supported the role of miR482, miR1512, and miR1515 with enhanced nodule numbers at the transgenic level, thus suggesting their role in nodule development in soybean. However, Wang et al. (2015) [136] demonstrated that overexpression of miR156 in transgenic plants caused inhibited nodule development in *Lotus japonicus*. Similarly, in common bean, overexpression of miR319 the target *TCP10* TF gene mRNA, which positively induces the action of the *LOX2* gene involved in jasmonic acid synthesis [141], stimulated the nodule development but decreased the rhizobial infection process [141].

Furthermore, to gain deeper insight into the role of miRNAs regulating nodulation and the symbiosis process, Sós-Hegedűs et al. (2020) [142] established and functionally validated the regulatory mechanism of the nodulation and symbiosis process through silencing of target NB-LRR genes by miR2118, miR2109, and miR1507 miRNAs in *Medicago truncatula*. During nodulation and symbiotic nitrogen fixation, the symbiotic bacteria upregulate miR2118, miR2109, and miR1507 miRNAs, at the cost of downregulating *NB-LRR* genes; consequently, the plant’s innate immunity is compromised during symbiosis in nodules [142] (see Figure 2). Recently, Tsikou et al. (2018) [187] and Gautrat et al. (2020) [131] suggested that miR2111 targeting *TOO MUCH LOVE* (encoding F-box/kelch-repeat protein), a nodulation suppressor, could enhance nodulation. However, the prevalence of rhizobial inoculation/infection and nitrate treatment reduced the level of miR2111s in leaves and roots, depending on the shoot-acting hypernodulation and aberrant root 1 (HAR1) receptor. Moreover, describing the fine-tuning and autoregulation mechanism of nodulation, Gautrat et al. (2020) [131] postulated that the Clavata3/Embryo surrounding region 12 (CLE12) and the CLE13 signalling peptides synthesized in roots act through HAR1/super numeric nodule (SUNN) receptors to negatively regulate the action of miR2111 [130]. This miR2111 otherwise favours root symbiotic nodulation under nitrogen-starved conditions by C-terminally encoded peptide (CEP) produced in root and acts in shoot through the compact root architecture 2 (CRA2) receptor. Likewise, Okuma et al. (2020) [130] confirmed the regulatory role of HAR1-dependent miR2111s produced from the *MIR2111-5* locus in shoots controlling root nodulation in *Lotus japonicus* using functional analysis.

Apart from these model legumes, three *A. hypogaea*-specific miRNAs, ahy-miR3508 (targeting gene encoding pectinesterase), ahy-miR3509, and ahy-miR3516, were identified; however, it is not known whether they participate in the nodulation process [108]. In common bean, genome-wide transcriptome analysis using Genome Analyzer IIx and degradome analysis identified 185 mature miRNAs and 181 targets for these identified miRNAs [100]. Functional characterization of selected miRNAs, viz., miRNov153 targeting uridine kinase (*Phvul.003 g180800*), miR319 targeting TCP TF family member (*Phvul.011 g156900*), and miR-Nov494 targeting aldehyde dehydrogenase (*Phvul.004G162200.1*), were upregulated, but their corresponding target genes were downregulated, indicating their significant involvement in controlling nodule development in common bean [100].

Furthermore, these miRNAs, an abundance of 21-nucleotide phased siRNAs derived from *PHAS* loci corresponding to protein coding genes *NB-LRRs*, were noted in soybean nodule [90] and in common bean nodule [100]. Likewise, evidence of circRNAs involved in nodule development and rhizobial symbiosis has been reported in common bean [188]. The authors suggested their role of acting as eTM and regulating the transmembrane transport and positive regulation of kinase activity during nodule development and the nitrogen fixation process. Recently, Tiwari et al. (2021) [189] and Hoang et al. (2020) [190] comprehensively discussed the interplay of various miRNAs impacting hormone signalling and regulating various regulatory genes during rhizobial infection, nodule organogenesis, and nitrogen fixation. A thorough understanding of various gene networks and their interplay with regulatory ncRNAs and precise function in controlling nodulation and related processes during the symbiosis process will further illuminate our insights into legume symbiosis at the molecular level involving ncRNAs.

## 11. Conclusions and Future Perspectives

The discovery of ncRNAs and their functional annotation have received considerable interest for investigating the underlying molecular mechanisms controlling various biological phenomena in legumes and opened a new avenue for improving traits of interest. As ncRNAs are dynamic, they are rapidly being discovered and functionally characterized in various plant species, including legumes [19]. However, the complete characterization of discovered ncRNAs at the functional level and their target gene(s) is limited to model legumes, viz., *M. truncatula*, *L. japonicus*, and soybean. Other legumes also need attention for the investigation of novel ncRNAs and their functions. Emerging approaches including powerful deep transcriptome sequencing technologies and advances in computational biology will facilitate the discovery of more ncRNAs and annotate their function. Moreover, emerging approaches of genome editing technology, viz., CRISPR/Cas9, will enable the functional characterization of novel ncRNAs (through loss-of-function/gain-of-function analysis) or manipulation of miRNAs causing the reprogramming of gene expression that controlling various traits of breeding importance with high precision [21,130,191]. Thus, the artificial manipulation of ncRNAs controlling various breeding traits could help us develop designer crops for sustaining global food security under predicted climate change scenarios.

## Figures and Tables

**Table 1:** List of published ncRNAs in legume plants regulating growth and development and biotic and abiotic stress responses.

Number of ncRNA	Crop	Genotype	Trait	Tissue	References
416 miRNAs	*M. truncatula*	Jemalong A17	Symbiosis and pathogenic interactions	Roots	[75]
100 novel candidate miRNAs	*M. truncatula*		Root and nodule development	*–*	[76]
201 individual miRNAs	*M. truncatula*	Jemalong	Heavy metal	Seedlings	[77]
326 known miRNAs and 21 new miRNAs	*M. truncatula*	Jemalong A17	Aluminium toxicity	Root apices	[78]
301 known miRNAs and identified 3 new miRNAs	*M. truncatula*	*–*	Ethylene response	Roots	[79]
26 novel miRNAs	*M. truncatula*	Jemalong	*–*	Leaves	[50]
385 conserved miRNAs and 68 novel miRNAs	*M. truncatula Medicago sativa*	Jemalong A17, Zhongmu-1	Salinity stress	Roots	[80]
876 miRNAs	*M. truncatula*	R108	Salinity	Seedlings	[59]
100 novel candidate miRNAs	*M. truncatula*	Jemalong A17	Root and nodule development	Roots	[76]
8 miRNAs	*M. truncatula*	Jemalong	*–*	Roots, shoots	[51]
219 novel *L. japonicus* micro RNAs	*Lotus japonicus*	Gifu wild-type	Epidermal and cortical signalling events	*–*	[81]
3030 long intergenic noncoding RNAs (lincRNAs), 275 natural antisense transcripts (lncNATs)	Soybean	Williams 82	Salinity	Roots	[82]
55 families of miRNAs	Soybean	Williams82	Nodulation	Roots	[49]
5372 circRNAs	Soybean	*–*	Developmental process	Stems, roots, mature leaves	[16]
537 known and 70 putative novel miRNAs	Soybean	KS4607, KS4313N	Soybean cyst nematode	Roots	[67]
71 miRNAs	Soybean	Williams 82	Salinity	Roots	[83]
364 + 21	Soybean	Hairbin xiaoheidou, Liaodou 10	Soybean cyst nematode	Roots	[74]
284 miRNAs	Soybean	Williams 82	Nodulation	Roots	[84]
120 miRNA genes	Soybean	Williams82	Root, nodule, organ development	Roots, stems, young leaves	[85]
362 known miRNAs	Soybean	No.116, No.84-70	Nitrogen stress	Roots, shoots	[86]
38+8 miRNAs	Soybean	Heinong44	Seed development	Seeds	[87]
6018 lincRNAs	Soybean	–	Various agronomic trait	Flower buds, unopened flowers, florescence, pods, seeds	[61]
46 lncRNAs	Soybean	MT72 and JN18	Fatty acid synthesis	Pods	[88]
158 novel miRNAs and 160 high-confidence soybean miRNAs	Soybean	NJCMS1A, NJCMS1B	Male sterility	Flower buds	[89]
500 loci generating phasiRNAs from PHAS loci	Soybean	Williams 82	Reproductive development	Anther and ovary tissues	[90]
2248 lincRNAs	Chickpea		Flower development	Vegetative tissues, shoot apical meristem, young leaves	[91]
59 novel miRNAs	Chickpea	ICC4958	Fusarium wilt, salinity	Roots	[68]
157 miRNA loci	Chickpea	ICC4958	Stress response	Leaves, inflorescence	[12]
440 conserved miRNAs + 178 novel miRNAs	Chickpea	ICC4958	Diverse cellular processes and metabolism	Leaves, stems, flower buds, young pods	[92]
651 miRNAs	Chickpea	C 214, Pb 7, ILC 3279, ICCV 05530, BC3F6	Aschochyta blight	Seedlings	[69]
113 +243 miRNAs	Chickpea	JGK3 and Himchana1	Seed size and development	Seeds	[93]
74 known and 26 novel miRNAs	Chickpea	*–*	Seed development	Seeds	[94]
3457 high-confidence lncRNAs	Chickpea	ICC4958, ICC1882, ICCV2, JG62	Drought and salinity	*–*	[66]
284 unique miRNAs	Chickpea	BGD72	Drought and salinity	Roots	[95]
114 miRNAs	Common bean			Leaves, flower, roots	[96]
422 miRNAs	Common bean		MYMIV	Leaves	[97]
68 miRNAs	Common bean		Nutrient deficiency and manganese toxicity stress	Leaves, roots, nodules	[60]
72 known and 39 new miRNAs	Common bean	SER16	Seed development	Seeds	[98]
28 miRNAs	Common bean	Negro Jamapa 81	Aluminium toxicity	Nodules	[99]
185 mature miRNAs	Common bean	Negro Jamapa, Pinto Villa	N_2_-fixing symbiotic nodules	Flowers, leaves, roots, seedlings	[100]
197 lncRNAs	Common bean	BAT93	Fruit development	Flowers, pods, seeds, leaves, roots, stems	[101]
16 conserved miRNAs	Common bean	Negro Jamapa, Pinto Villa	Different stress	*–*	[43]
1442+ 189 lncRNAs	Groundnut	Fenghua-1	Development, growth and stress tolerance	Roots, leaves, seeds	[102]
50,873 lncRNAs	Groundnut		Growth and development	15 different tissues	[103]
334 peanut miRNAs	Groundnut	Huayu 20	Pod rot		[104]
70 known and 24 novel miRNAs	Groundnut	Luhua-14	Pod development	Gynophores	[105]
126 known miRNAs + 25 novel peanut	Groundnut		Development	Leaves, stems, roots, seeds	[56]
18 miRNAs	Groundnut		Disease resistant proteins, auxin responsive proteins	*–*	[106]
1,082 miRNAs	Groundnut	8106, 8107	Seed expansion	Seeds	[107]
32 miRNAs	Groundnut		Nodule development	Nodules	[108]
29 known and 132 potential novel miRNAs	Groundnut	Baisha1016	Ca deficiency	*–*	[109]
347 circRNAs	Groundnut	RIL 8106, RIL 8107	Seed development and size	*–*	[110]
9388 known and 4037 novel lncRNAs	Groundnut	Huayou 7, Huayou 4	Seed development	Seeds	[111]
617 mature microRNAs	Cowpea		Cowpea severe mosaic virus	Leaves	[112]
17 new miRNAs	Cowpea	Dan lla, Tvu7778	Drought	Leaves, roots	[113]
157 miRNA genes	Cowpea	CB46, IT93K503-1	Drought	Leaves	[55]
18 miRNAs	Cowpea		Salinity stress	Roots	[114]
616 mature miRNAs + 3919 lncRNAs	Pigeonpea	*–*	*–*	–	[115]
3919 lncRNAs	Pigeonpea		*–*	–	[115]
3019 lncRNAs and 227 miRNAs	Pigeonpea	Asha	Seed and pod development	Seeds, pods	[57]
298 upregulated and 395 downregulated 284 upregulated and 243 downregulated	Faba bean	Hassawi-3 ILB4347	Salinity	Leaves	[116]
66 miRNAs	Urd bean			Leaves, stems, roots	[117]
56miRNAs	Narrow-leafed lupin	Tanjil	Seed development	Stems, leaves, seeds	[118]
167 miRNAs	White lupin		Phosphate deficiency	Roots, stems, leaves	[119]
394 known and 28 novel miRNAs and 316 phased siRNAs	Yellow lupine	Taper	Floral development and abscission	Flowers	[120]
143 and 128	Lathyrus	IC-143067	Drought	*–*	[121]
47 and 44 miRNAs	Alfalfa		Phosphorus deficiency	Roots, shoots	[13]
371 circRNAs	Soybean	Bogao, Nannong 94156	Phosphorus deficiency	Roots	[14]

**Table 2:** Role of ncRNAs controlling abiotic and biotic stresses and other growth and development in legume plants with possible molecular mechanisms involved.

Name of ncRNA	Crop	Trait/Stress	Target Gene(s)/Protein Coding Gene(s)	Function	References
miR408	Chickpea	Drought	*DREB*	Overexpression represses plantacyanin encoding genes and controls DREB regulation under water stress	[122]
16 drought-responsive miRNAs	Common bean	Drought	TFs and protein kinases	Control drought stress by targeting various TFs and protein kinases	[123]
6 downregulated and 6 upregulated miRNAs	Soybean	Drought	Auxin signalling, plantacyanin, Cu/Zn superoxide dismutases	Control drought stress by targeting auxin signalling, plantacyanin and Cu/Zn superoxide dismutases encoding genes	[124]
44 drought-responsive miRNAs	Cowpea	Drought	Zinc finger family protein, serine/threonine protein kinase	Involved in development and stress response	[55]
vun-miR5021, vun-miR156b-3p, vun-miR5021, vun-miR156b, vun-miR156f	Cowpea	Drought	Kelch repeat-containing F-box protein, *CPRD86*, *P5CS*, *multicystatin* gene, and glutathione reductase	Induce genes *PLD* (phospholipase D), *APX* (ascorbate peroxidase) and *P5CS* (delta 1-pyrroline-5-carboxylate synthase) under stress	[113]
miR162, miR164, miR319, miR403, miR828, miR160a, miR160b, miR171e, vun_cand015, vun_cand033, vun_cand048, miR171b, miR171d, miR2111b, miR390b, and miR393, vun_cand001, vun_cand010, vun_cand041, vun_cand057	Cowpea	Drought	ARF10, ARF8, zinc finger protein, basic-helix-loop-helix (bHLH), TF leucine-rich repeat transmembrane protein kinase, pentatricopeptide repeat-containing protein	Involved in development and stress response	[55]
miR398a/b, miR408	Pea	Drought	Copper superoxide dismutase, *CSD1*	Reduce oxidative stress	[125]
lsa-miR169b, lsa-miR1508a, lsa-miR319a, lsa-miR156a, lsa-miR398b, lsa-miR396d, lsa-miR166b, lsa-miR390a, lsa-miR167b, lsa-miR186, lsa-miR786, lsa-miR897, lsa- miR969 and lsa-miR1361, miR397, miR398, miR164, miR399	Lathyrus	Drought	*F*-*box*, *U*-*Box* or protein coding genes involved in proline, betain, and osmolyte biosynthesis pathway	Induce osmo-protective compounds under stress	[121]
	Chickpea	Drought and salinity	*LACCASE4*, *COPPER SUPEROXIDE DISMUTASE* (Cu-SOD), *NAC1* and *PHO2/UBC24*	Increase lateral root formation and improves uptake of K^+^ under salinity stress	[95]
*MIR2119 and MIR398a*	Common bean	Drought	*ALCOHOL DEHYDROGENASE 1* (*ADH1*) and *COPPER-ZINC SUPEROXIDE DISMUTASE 1* (*CSD1*)	By reducing oxidative stress	[45,48]
pvu-miR2118	Common bean	Drought	–	Controls drought stress	[43]
miR169, miR398a/b and miR408	*M. truncatula*	Drought stress	Copper proteins COX5b, copper superoxide dismutase, and plantacyanin		[58]
miR172a	Soybean	Salinity	*Glyma.10G116600*, *Glyma.02G087400*, *Glyma.13G329700*, *Glyma.12G073300*, *Glyma.15G044400*, *Glyma.11G053800*, *AP2*/*EREBP*-type TF gene *SSAC1*, thiamine biosynthesis gene *THI1*	Induction cleaves mRNA transcripts of salt-suppressed AP2 domain-containing genes increasing expression of thiamine biosynthesis gene *THI1* and resulting salinity tolerance	[126]
18 conserved miRNAs	Cowpea	Salinity	15 target genes	Control plant development and root growth under stress conditions by targeting various TF genes viz., *SBP*, *ARF*, *SPL*, *TCP*, *NFY*, and *AP2*	[114]
miR156_1, miR156_10, car-miR008, car-miR011, car-miR015	Chickpea	Salinity	Squamosa promoter-binding protein	Target protein-encoding gene to control salinity stress	[68]
lncRNA TCONS_ 00097188, TCONS_00046739, TCONS_00100258, TCONS_ 00118328, TCONS_00047650, lncRNA TCONS_ 00020253, TCONS_00116877	*Medicago truncatula*	Salinity	*Medtr6g006990*, *cytochrome P450*, *Medtr3g069280*, *Medtr1g081900*, *Medtr7g094600*	Upregulate various gene expression contributing to salinity stress adaptation	[15]
TCONS_ 00292946, TCONS_00176941, TCONS_00011551	Groundnut	Salinity	–	Control salinity stress tolerance	[102]
pvu-miR159.2	Common bean	Salinity	–	–	[43]
miR160, miR156/157, miR159, miR169, miR172, miR408	Cowpea	Salinity stress	Auxin response factor (ARF), squamosa promoter-binding protein (SBP), TCP family transcription factor, CCAAT-binding transcription factor (CBF), PHAP2B protein, APETALA2 protein (AP2), Basic blue copper protein/Plantacyanin	Target TFs and control salinity stress	[114]
lncRNA MtCIR1	*Medicago truncatula*	Cold stress	*MtCBF* genes	Controls cold tolerance	[127]
soy_25	Soybean	Seed development	*Glyma05g33260*	Controls seed development	[87]
gma-miR168	Soybean	–	*Glyma16g34300*		
miR167, miR399, miR156, miR319, miR164, miR166, miR1507 and miR396	Narrow leaf lupin	Seed development	*GROWTH-REGULATING FACTOR* (GRF) TF, SBP-box transcription factors, MYB transcription factors, Zinc finger domain proteins, molybdate transporter 1, calcium-transporting ATPase 8, TMV resistance protein N, lysine-specific demethylase JMJ16, nudix hydrolase protein	Target TF (Class III HD-Zip, NAC) related to seed development process	[118]
ahy_novel_miRn1 to ahy_novel_miRn132, miR3509, miR3511, and miR3512, miR159 and miR167, miR3514, miR3518	Groundnut	Ca deficiency driven embryo abortion	*TCP3*, *AP2*, *EMB2750*, *GRFs*, *HsfB4*, *DIVARICATA*, *CYP707A1*, *CYP707A3*	Regulate embryo abnormality under Ca deficiency by modulating the target genes	[109]
miR_18, miR_6, miR_11, miR_29, miR_6, miR_38, miR_6, pvu-miR399a, miR_18, miR_33, miR_16, pvu-miR156i	Common bean	Seed development	DEHYDRIN FAMILY PROTEIN (RAB18), DEAD BOX RNA HELICASE (PRH75) CESA3, LEUCINE-RICH PROTEIN KINASE FAMILY PROTEIN, PRH75, MEE9, EM1, PHO2, RAB18, PROTEIN KINASE SUPERFAMILY PROTEIN, DUF827, and SPL2	Regulate these genes during various stages of seed development, viz., seed filling, maturation, and dormancy	[98]
XR_001593099.1, MSTRG.18462.1, MSTRG.34915.1, MSTRG.41848.1, MSTRG.22884.1, MSTRG.12404.1, MSTRG.26719.1, MSTRG.35761.1, MSTRG.20033.1, MSTRG.13500.1, MSTRG.9304.1	Groundnut	Seed development	*XM_016114848.1*, *XM_ 016087708.1*, *XM_016309191.1*, *XM_ 016324297.1*, *XM_016327810.1*, *XM_016116309.1*, *XM_ 016335443.1*, *XM_ 016310265.1*, *XM_ 016091385.1*	Regulate groundnut seed development by modulating the target genes encoding MADS-box transcription factor 23-like, protein transport protein sec31-like, squamosa promoter-binding-like protein 14	[111]
Ca_linc_0051 and Ca_linc_0139	Chickpea	Flower development			[91]
miR156/157, miR164, miR167, miR1088, miR172, miR396	Groundnut	Pod development	*SPL*, *NAC*, *PPRP*, *AP2*, *GRF*	Control pod development	[105]
Cc_lncRNA-2830	Pigeonpea	Pod development	*miR160h- Auxin responsive factor-18*	Upregulates Cc_lncRNA-2830, sequesters miR160h promoting expression of auxin responsive factor-18 and helps in pod formation	[57]
gma-miR156b and gma-miR156f, gma-miR162a, gma-miR162b, gma- miR162c, gma-miR399d, gma-miR399e, gma- miR399f gma-miR399g	Soybean	Male sterility	MADS-box transcription factor, NADP-dependent isocitrate dehydrogenase, 6-phosphogluconate dehydrogenase, NADH-ubiquinone oxidoreductase	Target these genes and cause programmed cell death, ROS toxicity and energy deficiency	[89]
lncRNA MSTRG.45502.1, lncRNAs MSTRG.40968.1	Soybean	Lipid metabolic processes	*XM_003538388.3,XM_006588497.2 00,061*		[88]
miR393j-3p	Soybean	Nodule development	*Early Nodulin 93* (*ENOD93*)	Targets *Early Nodulin 93* (*ENOD93*) gene and regulates nodule formation	[84]
gma-miR2606b, gma-miR4416	Soybean	Nodule development	*Mannosyl- oligosaccharide 1*, *2-alpha-mannosidase*, *Rhizobium-induced peroxidase 1* (RIP1)-like peroxidase gene	Target these genes to positively and negatively regulate the nodulation process	[128]
miR482, miR1512, miR1515	Soybean	Nodule development	*Gm12g28730*, *Gm17g04060*, *Gm04g05920*, *Glyma09g27690*	Regulates nodulation process	[129]
miR2111	*Lotus japonicus*	Nodulation	*TOO MUCH LOVE*, a nodulation suppressor	Low expression after rhizobial infection relying on shoot-acting HYPERNODULATION ABERRANT ROOT FORMATION1 (HAR1) receptor	[130]
miR2111	*M. truncatula*	Nodulation and symbiosis	*Too Much Love 1*, *Too Much Love 2*	Positively controls root symbiotic nodulation, which is systemic from shoots and depends on the CRA2 receptor	[131]
MIR166	*M. truncatula*	Root and nodule development	*Class-III HD-ZIP* genes	Overexpression reduced the number of symbiotic nodules and lateral roots	[132]
microRNA169	*M. truncatula*	Nodule development	*MtHAP2-1*	Regulates *MtHAP2-1* gene controlling symbiotic nodule formation	[133]
ahy-mi399, ahy-miR159, ahy-miR3508	Groundnut	Nodule infection	*Pectinesterase gene*	Regulate nodulation development process	[108]
miRNA 172	Soybean	Nodulation	AP2 transcription factor	Controls miR172 expression and regulates AP2 TF activity	[134]
miRNA 172c	Soybean	Nodulation	*Nodule Number Control1*	Controls nodule formation by repressing its target gene	[135]
miRNA156	*Lotus japonicus*	Nodulation	*ENOD* genes, *SymPK*, *POLLUX*, *CYCLOPS*, *Cerberus*, *and Nsp1*, *SPLs*	Represses downstream target *SPLs* and other nodulation genes	[136]
*MtENOD40*	*M. truncatula*	Nodule development	–	Regulates re-localization of proteins	[38]
*GmENOD40*	Soybean	Nodule development	–	Regulates re-localization of proteins	[137]
miR156e, miR156g, miR167b	*M. truncatula*	Symbiosis signals	Induced by Myc-LCO and repressed by Nod signals		[75]
miR172a	*Lotus japonicus*	Epidermal infection during symbiosis	APETALA2-type (AP2) transcription factors	Targets AP2 TF and regulates bacterial symbiosis	[81]
miR171 isoform, miR397	*Lotus japonicus*	N_2_ fixation	Laccase copper protein family, Nodulation Signalling Pathway2	Respond to symbiotic infection and nodule function	[138]
miR396	*M. truncatula*	Root growth and mycorrhizal associations	Growth-regulating factor genes (*MtGRF*) and two *bHLH79*-like target genes	Regulates root growth and mycorrhizal associations	[139]
miR171h	*M. truncatula*	Mycorrhizal colonization	*NSP2*	Targets *NSP2* and modulates mycorrhizal colonization	[140]
miR1507, miR2118, miR2119, miR2199	*M. truncatula*	Pathogen infection	TIR-NBS-LRR proteins targeted by miR2118 auxin response factor (ARF)	miRNA-mediated plant defence response	[51]
miR319d	Common bean	Rhizobium N_2_ fixation	*TCP10* (Phvul.005G067950)		[141]
miR1507, miR2109, miR2118	*M. truncatula*	Nodulation and symbiosis	*NB-LRR* genes	Suppress activity of *NB-LRR* genes and allow nodulation process	[142]
ENOD40	Soybean	Nodule development	–	–	[137]
ENOD40	and *M. truncatula*	Nodule development	-	–	[38]
617 mature microRNAs	Cowpea	Cowpea severe mosaic virus	Kat-p80, DEAD-Box, GST, and SPB9	Involved in defence response to CSMV	[112]
vun-miR156a, vun-miR156b, vun-miR156b-3p, vun-miR156b-5p, vun-miR156f, vun-miR156 g, vun-miR157d, vun-miR2610a, vun-miR2673b, vun-miR5021			Ted2 protein gene, Glutathione reductase, R3H domain protein gene, P5CS, Phosphoribosylpyrophosphate amidotransferase, 5-aminoimidazole ribonucleotide carboxylase, R3H domain protein gene, Ted2 protein, 5-aminoimidazole ribonucleotide carboxylase, Vigna unguiculata extensine-like protein 3, Aspartic proteinase, CPRD86		
miR156, miR159, miR160, miR166, miR398, miR1511, miR1514, miR2118, and novel vmu-miRn7, vmu-miRn8, vmu-miRn13, vmu-miRn14	Urdbean	MYMIV	NB-LRR, NAC, MYB, Zinc finger, CCAAT-box transcription factor, fructose 2-6 bisphosphate, HDZIP protein	Participate in defence/immune response to MYMIV	[143]
miR530	Chickpea	Fusarium wilt infection	Zinc knuckle- and microtubule-associated proteins	Regulates plant defence against pathogen attack	[68]
miR166	Chickpea	–	HD-ZIPIII transcription factor	–	[68]
car-miRNA008	Chickpea	Natural defence	*Chalcone synthase* (*CHS*) gene	Regulates plant defence against pathogen attack	[68]
car-miR2118, car-miR5213	Chickpea	Defence response	TIR-NBS-LRR	Regulate plant defence against pathogen attack	[68]
miR156, miR159, miR160, miR162, miR164, miR168, miR172, miR393, miR408	Chickpea	Stress response and development processes	SPB factor, MYB transcription factor, ARFs, DCL1, HD-Zip, Arg onaute 1, AP2, F-box protein, plantacyanin	Target superoxide dismutases, plantacyanin, laccases and F-box proteins genes during stress	[12]
ahy-miR396e-5, ahy-miR3509-5p, ahy-miR166f, ahy-miR159b	Groundnut	Pod rot	*c39419_g1_i1*, *c40055_g1_i3*, *c31393_g1_i1*, *c41016_g4_i1*	Related to defence response	[104]
miR482b-3p, miR159k-3p, nov_miR66, miR171, miR162, miR167c, miR171b	Chickpea	Ascochyta blight resistance	NBS-LRR, PR protein, serine-threonine kinase, PPR protein, Dicer-like gene (*Ca_01367*), Dof zinc finger (Ca_19433), ERF (*Ca_00359*) gene	Produce pathogenesis-related protein, ROS activity, cell wall synthesis, hormone synthesis, R gene activation	[69]
miR171, miR159, miR399, miR398, miR408, miR9750, miR2119, miR1512	Soybean	Rootknot nematode	ATPase, Glycosyl hydrolases, multicopper oxidase, SOD, peroxidase, Glucose-6-phosphate dehydrogenase encoding genes	Regulate PR genes, oxidative stress and defence response	[67]
miR156/157, miR164, miR167 and miR1088, miR172, miR396	Groundnut	–	*SPL*, *NAC*, *PPRP*, *AP2 GRF*	Control seed development	[105]
miR157, miR156, miR170, miR172, miR319, miR398, pvu-miR159.2, pvu-miR2118, gma-miR1508, gma-miR1526, gma-miR1532, miR160, miR397, miR399, miR408, pvu-miR1509, pvu-miR1514a	Common bean	Manganese toxicity	–	Upregulated miR157, miR156, miR170, miR172 and downregulated pvu-miR2118, gma-miR1508, gma-miR1526, and gma-miR1532, etc.	[60]
miR2681, miR2708, miR2687	*M. truncatula*	Mercury tolerance	*TIR-NBS-LRR*, *TC114805*, xyloglucan endotransglucosylase/ hydrolase (XTH)	XTH helps in cell wall development under heavy metal stress	[77]
Gm03circRNA1785	Soybean		gma-miR167c and *GmARF6* and *GmARF8*		[16]
PDIL1, PDIL2, PDIL3	*M. truncatula*	Phosphate starvation	*MtPHO2*, *Medtr1g074930*	Regulate phosphate uptake	[17]
miR399	Common bean	Phosphorus deficiency	*PvHAD1*		[144]
gma-miR156b/6f-5p, gma-miR396b∼g-5p, gma-miR5372-5p, gma-miR159d-3p, gma-miR396b∼g-5p	Soybean	Nitrogen deficiency	*Glyma07g31580*, *Glyma05g20930*, *Glyma06g18790*, *Glyma09g02600*, *Glyma05g23280*, *Glyma07g05550*, *Glyma16g02090*, *Glyma17g16750*, *Glyma19g44930*, *Glyma15g08010*, *Glyma19g01200*	Play role in protein degradation	[86]
miR399, miR398, miR156, miR159, miR164, miR168, miR172, miR393, miR408	Alfalfa	Phosphate starvation	*Phosphate transporter*, *Copper chaperone for SOD*, *Squamosa promoter-binding-like* (*SPL*), *MYB TF*, *auxin response factor* (*ARF*), *GRAS*, *MATE*	Regulate phosphate uptake	[13]
circ_000232	Soybean	Phosphorus deficiency	*Glyma.13G117700*	Regulates P use efficiency	[14]

## Data Availability

Not applicable.
